# [Retracted] SRC‑like adaptor protein negatively regulates Wnt signaling in intrahepatic cholangiocarcinoma

**DOI:** 10.3892/ol.2024.14692

**Published:** 2024-09-23

**Authors:** Yong Wang, Xinxin He, Yangnian Wei, Ling Liu, Wen Wang, Nianfeng Li

Oncol Lett 17: 2745–2753, 2019; DOI: 10.3892/ol.2019.9901

Following the publication of this paper, it was drawn to the Editor's attention by a concerned reader that the cell migration and invasion assay data shown in Fig. 5A and B on p. 2751 had apparently already been published previously in a number of different research articles written by different authors at different research institutes. Owing to the fact that the contentious data in the above article had already been published prior to its submission to *Oncology Letters*, the Editor has decided that this paper should be retracted from the Journal. The authors were asked for an explanation to account for these concerns, but the Editorial Office did not receive a reply. The Editor apologizes to the readership for any inconvenience caused.

